# Role of Specialized Pro-resolving Mediators in Reducing Neuroinflammation in Neurodegenerative Disorders

**DOI:** 10.3389/fnagi.2022.780811

**Published:** 2022-02-17

**Authors:** Jana Ponce, Arzu Ulu, Corrine Hanson, Erin Cameron-Smith, John Bertoni, Jenna Wuebker, Alfred Fisher, Ka-Chun Siu, Vivien Marmelat, Jiri Adamec, Danish Bhatti

**Affiliations:** ^1^Division of Medical Nutrition Education, College of Allied Health Professions, University of Nebraska Medical Center, Omaha, NE, United States; ^2^Division of Biomedical Sciences, School of Medicine, University of California, Riverside, Riverside, CA, United States; ^3^Department of Neurological Sciences, College of Medicine, University of Nebraska Medical Center, Omaha, NE, United States; ^4^Department of Pharmaceutical and Nutrition Care, Nebraska Medicine, Omaha, NE, United States; ^5^Department of Internal Medicine, College of Medicine, University of Nebraska Medical Center, Omaha, NE, United States; ^6^Department of Biomechanics, College of Education, Health, and Human Sciences, University of Nebraska - Omaha, Omaha, NE, United States; ^7^Department of Biochemistry, College of Arts and Sciences, University of Nebraska - Lincoln, Lincoln, NE, United States

**Keywords:** neurodegenerative disorder, Parkinson’s disease, Alzheimer’s disease, neuroinflammation, specialized pro-resolving lipid mediator (SPM), omega-3 fatty acids, omega-6 fatty acid, polyunsaturated fatty acids

## Abstract

Alzheimer’s disease (AD) and Parkinson’s disease (PD) are neurodegenerative disorders that affect millions of individuals worldwide. As incidence of these conditions increases with age, there will undoubtedly be an increased prevalence of cases in the near future. Neuroinflammation is a hallmark in the development and progression of neurodegenerative diseases and prevention or resolution of chronic neuroinflammation may represent a novel approach to treatment. The present review highlights the potential of the anti-inflammatory and pro-resolving effects of polyunsaturated fatty acid (PUFA)-derived mediators (Specialized Pro-resolving Mediators—SPM) in neurodegenerative disorders. PUFA-derived SPM are biosynthesized in response to chemicals produced from acute inflammatory responses. Preclinical studies from both AD and PD models suggest a dysregulation of SPM and their receptors in neurological disorders. Decreased SPM may be due to inadequate substrate, an imbalance between SPM and pro-inflammatory mediators or a disruption in SPM synthesis. SPMs hold great promise for neuroprotection in AD by altering expression of pro-inflammatory genes, modulating macrophage function, serving as a biomarker for AD status, and promoting resolution of neuroinflammation. In PD, data suggest SPM are able to cross the blood-brain barrier, inhibit microglial activation and decrease induced markers of inflammation, possibly as a result of their ability to downregulate NFκB signaling pathways. Several *in vivo* and *in vitro* studies suggest a benefit from administration of SPMs in both neurodegenerative disorders. However, extrapolation of these outcomes to humans is difficult as no models are able to replicate all features of AD or PD. Minimal data evaluating these PUFA-derived metabolites in humans with neurodegenerative disorders are available and a gap in knowledge exists regarding behavior of SPM and their receptors in patients with these conditions. There is also large gap in our knowledge regarding which lipid mediator would be most effective in which model of AD or PD and how dietary intake or supplementation can impact SPM levels. Future direction should include focused, translational efforts to investigate SPM as an add-on (in addition to standard treatment) or as standalone agents in patients with neurodegenerative disorders.

## Introduction

Millions of people worldwide are living with neurodegenerative disorders, making these conditions a significant public health problem. Today, 5 million suffer from Alzheimer’s disease (AD) and 1 million Americans suffer from Parkinson’s disease (PD) (Checkoway et al., 2011). Neurodegenerative disorders are associated with substantial mental, physical, and social impairment, which can lead to decreased quality of life. In addition, these conditions are a significant economic burden to society with a yearly direct cost of hospitalizations and medications of $25.4 billion (Kowal et al., 2013). Increased prevalence of neurodegenerative diseases is, in part, due to the aging population (Cova et al., 2017). While genetic and environmental factors for many neurodegenerative disorders have been identified, age remains the primary risk factor (Hou et al., 2019). By 2060, the U.S. census bureau estimates the number of Americans ages 65 and older is projected to nearly double from 52 million in 2018 to 95 million. As incidence of neurodegenerative diseases increases with age, there will undoubtedly be an increased prevalence of cases. In fact, a study by the Parkinson’s Foundation estimates the number of PD cases will rise to 1.24 million by 2030 (Marras et al., 2018). Without a known cure, prevention and the slowing of progression of these diseases in an aging population are paramount.

Neuroinflammation is a hallmark in aging as well as in the development and progression of neurodegenerative diseases (Carson et al., 2006; Wang et al., 2015a; Chen et al., 2016; Rai et al., 2016; Niranjan, 2018). The term refers to a variety of immune responses to an initial stimulus in the central nervous system, resulting in increased levels of cytokines, chemokines, and free radical production. Degree of neuroinflammation depends on the primary insult, context and duration (DiSabato et al., 2016). An efficient pro-resolution mechanism is essential for tissue homeostasis and the prevention of chronic neuroinflammation, which can lead to recruitment of immune cells, edema, tissue damage, and the activation of microglial cells (Serhan et al., 2000; Serhan, 2014). Microglia activation is an early feature that occurs in almost any neuronal physiology change. Activation of microglial cells has been shown to occur early in the pathological process of AD and PD (McGeer et al., 1988; Barcia et al., 2003; Croisier et al., 2005; Leng and Edison, 2021) and continues through the late stages. Activated microglial cells are responsible for the production of many cytokines including tumor necrosis factor-α (TNF-α), inter-leukin-1β (IL-1β), and chemokines. Additionally, microglial activation results in the production of vascular cell adhesion molecule-1 (VCAM-1) and intracellular cell adhesion molecule-1 (ICAM-1) which lead to dopamine neuron apoptosis or death (Benner et al., 2008; Wypijewska et al., 2010; Phani et al., 2012). Chronic microglia activation and neuroinflammation resulting in pro-inflammatory cytokines are associated with motor and cognitive function loss (Dinarello, 2000; Franceschi et al., 2007; Gyengesi et al., 2019).

Inhibiting pro-inflammatory mediators, reducing microglial activation and improving neuronal survival could be effective therapeutic strategies to mitigate the development and progression of neurodegenerative disorders. Interestingly, polyunsaturated fatty acids (PUFAs) have recently been found to serve as substrates for the biosynthesis of specialized pro-resolving lipid mediators (SPM) that promote the endogenous resolution of inflammation (Bannenberg and Serhan, 2010; Serhan, 2014). This discovery has prompted investigations into the ability of SPM to decrease neuroinflammation and downstream effects such as motor and cognitive decline. This review aims to summarize key research findings of these fatty acid (FA)-derived mediators as potential prevention or treatment for neuroinflammation in neurodegenerative disorders.

## Polyunsaturated Fatty Acids, Specialized Pro-Resolving Lipid Mediators and Aging

PUFAs are long-chain fat molecules that have more than one unsaturated carbon bond. Humans are unable to synthesize these long chain fatty acids and are required in the diet to prevent clinical deficiency. PUFAs are often divided into two major classes: omega (n)-3 and (n)-6 FAs. Alpha-linolenic acid (ALA), eicosapentaenoic acid (EPA), and docosahexaenoic acid (DHA) are the primary n-3 FAs. ALA (18:3) is present in plant oils such as flaxseed, soybean, and canola oils and can be converted to EPA and DHA in the liver (Beadle et al., 1965; Astorg, 2004). However, with a very low conversion rate of 0–4%, ingesting EPA and DHA directly from foods is the best way to increase levels in the body (Plourde and Cunnane, 2007). Major forms of n-6 FAs include linoleic acid (LA) (18:2) and arachidonic acid (ARA) (C20:4). LA is derived from vegetable and meat sources while ARA is metabolized from LA. Low n-3 intake is a common characteristic of the Western diet due to low intakes of foods such as fatty fish and nuts (Simopoulos, 2002). N-6 FAs are consumed at a disproportionately high level in a standard Western diet compared to n-3 due to high intakes of meat, processed foods, soy, and corn oils (Astorg, 2004).

**FIGURE 1:**
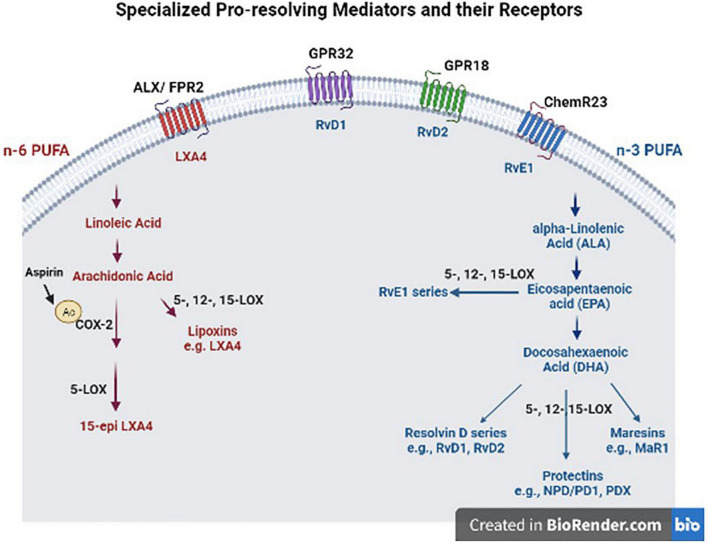
Specialized pro-resolving mediators (SPM) and their receptors. These SPM are derived from omega (n)-3 and n-6 polyunsaturated fatty acids (PUFAS) in response to chemicals produced from acute inflammatory responses. ALX/FPR2, formyl peptide receptor 2; LXA4, lipoxin A4; GPR32, G protein-coupled receptor 32; RvD1, resolvin D1; GPR18, G protein-coupled receptor 18; RvD2, resolvin D2; ChemR23, chemerin receptor 23; RvE1, resolvin E1; COX-2, cyclooxygenase-2; LOX, lipoxygenase; MaR1, maresin 1; NPD/DP1, neuroprotectin/protectin D1.

Beneficial effects of n-3 FA, such as DHA and EPA have been well-recognized in brain health (Alessandri et al., 2004; Virtanen et al., 2013). They regulate gene expression and signaling pathways involved in neurotransmitter release, inflammation, and immunity (Basselin et al., 2012; Orr et al., 2013). N-3 FAs compete with their n-6 counterparts such as ARA as substrates for enzymes (cytochrome P450, COX, LOX) that generate numerous bioactive fatty acids, namely “eicosanoids,” which regulate inflammation and immunity. ARA is released from phospholipid membranes with the action of phospholipase 2 (PLA2) in response to inflammation; and *in vitro* studies suggest that different types of PLA enzymes have different preferences for DHA vs. ARA as a substrate (Mouchlis et al., 2018). In support of these observations, iPLA2 KO mice display reduced DHA metabolism and increased tendency to neuroinflammation, while iPLA2 is highly expressed in the brain (Yang et al., 2019). This is of importance, because DHA is the most abundant n-3 FA in the brain and DHA metabolism-generating bioactive lipid mediators not only have anti-inflammatory effects, but also have effects in resolution of inflammation (Serhan et al., 2004, 2015). Moreover, n-3 FA show their beneficial effects, in part, through their bioactive metabolites, SPMs, that are involved in resolution of inflammation and tissue repair (Levy and Serhan, 2014). During acute inflammation, ARA-derived metabolites such as prostaglandins are produced to recruit neutrophils to the inflammation site. This process reaches homeostasis by a mechanism involving some prostaglandin metabolites and activation of anti-inflammatory cytokines (Buckley et al., 2014). To further promote resolution of inflammation, SPMs are biosynthesized with a process called “lipid mediator class switching” which temporarily switches pro-inflammatory metabolite biosynthesis to SPM production (Levy et al., 2001; Levy and Serhan, 2014). SPM derived from n-3 include the resolvin D (RvD)-, maresin (MaR)-, and protectin- series from DHA and the resolvin E (RvE)- series from EPA, while those derived from the n-6, ARA, include leukotrienes and lipoxins (Figure 1) (Ryan and Godson, 2010; Serhan et al., 2011). This process stops monocyte and PMN recruitment and activates the tissue repair process by recruiting M2-like macrophages and regulatory T-cells.

SPM are biosynthesized in response to chemicals produced from acute inflammatory responses. However, published studies agree that SPM levels are decreased in animals and humans with PD and AD despite chronic inflammatory insult (Zhu et al., 2016; Krashia et al., 2019). Decreased SPM may be due to inadequate EPA, DHA, or ARA substrate, an imbalance between SPM and pro-inflammatory mediators or a disruption in SPM synthesis. Several epidemiological studies suggest that increased n-3 PUFA intake is associated with decreased risk or improved outcomes in neurodegenerative disorders (de Lau et al., 2006; Aden et al., 2011; Zhang et al., 2016). However, intake of n-3 is low in a standard Western diet pattern and lower serum levels have been significantly associated with age (Strike et al., 2016). While proposed dietary reference intakes (DRI) for combined EPA and DHA are 250–500 mg per day (Harris et al., 2009), data from the National Health and Nutrition Examination Survey (NHANES) show intake of DHA and EPA of adults 51 + were 63 ± 2 and 23 ± 1 mg/day, respectively (Papanikolaou et al., 2014). Decreased intake and inadequate serum levels of n-3 may be even lower in the elderly and patients with neurodegenerative disorders due to dysphagia, anorexia, and feeding difficulties. Deprivation of n-3 has been shown to decrease levels of DHA, increase markers of brain AA, and increase nitric oxide (NO) and lipid peroxide levels in the brain (Orr and Bazinet, 2008; Cardoso et al., 2014). This phenomenon may be due, in part, to failed resolution of inflammation due to inadequate SPM production.

## Specialized Pro-Resolving Lipid Mediators in Alzheimer’s Disease

The leading cause of dementia worldwide, AD is a progressive neurodegenerative disease that leads to a decline in cognitive function. The amyloid cascade is the most accepted hypothesis to explain AD pathogenesis with the main hallmarks of AD being neuritic plaques composed of amyloid beta (Aβ) peptides and neurofibrillary tangles (NFTs) made up of hyperphosphorylated microtubule-associated protein (p-tau) (Iqbal et al., 2010; Barage and Sonawane, 2015). Neuroinflammation is a common feature in AD evidenced by elevated plasma and postmortem levels of pro-inflammatory cytokines as well as significant dysfunction in inflammation resolution pathways (Wang et al., 2015c). While there is a potential benefit of NSAIDs for modulating neuroinflammation in psychiatric diseases (Astorg, 2004), epidemiological studies in neurodegenerative disorders are inconclusive. Given that NSAIDs target early phase of inflammation and can halt inflammation resolution by preventing the biosynthesis of n-3 and n-6 fatty acid-derived SPMs, alternative options appear necessary.

Several studies investigated the dysregulation of inflammation resolution in neurodegenerative diseases. A study by Wang et al. (2015b) compared SPM levels in postmortem cerebrospinal fluid (CSF) (15 AD patients) and brain tissues (10 AD patients) of AD patients, patients with mild (20 patients, MCI) and subjective (21 patients) cognitive impairment (SCI) and non-demented control subjects. This study found lower CSF levels of lipoxin A4 (LXA4, an arachidonic acid derived SPM) in AD patients as compared to MCI and SCI patients, while no significant differences were observed for the DHA-derived RvD1. However, the two SPMs showed a strong positive correlation with each other as well as with mini-mental state examination test, suggesting that SPMs are important in cognition. Similarly, ARA-derived LXA4 and leukotriene B4 (LTB4), and DHA-derived MaR1 levels were lower in the hippocampus of AD patients as compared to control subjects, which was confirmed with both ELISA and LC/MS/MS analysis in this study. These findings were consistent with the heightened levels of the pro-inflammatory prostaglandins in AD brains as compared to control subjects. This study also showed increased levels of SPM receptors, ALX and FPR2 (LXA4 and RvD1 receptors, respectively), and the EPA-derived RvE1 receptor, chemerin receptor 23 (ChemR23) on both glial cells, mainly astrocytes and neurons, in the hippocampus of AD brains as compared to control subjects. However, no correlation was observed between ALX/FPR2 or LXA4/RvD1 levels and tau proteins. Heightened SPM receptor levels were also accompanied by increased hippocampal levels of 15-LOX2, a key enzyme in LXA4 biosynthesis; PPARγ, a nuclear receptor involved in SPM action; and reduced anti-inflammatory cytokine IL-10 in AD brains as compared to controls. Many PUFA-derived lipid mediators are ligands for PPARγ, which is anti-inflammatory and is involved in adipogenesis. Overall, this study showed significant dysregulation of SPMs and an attempt at inflammation resolution to compensate for the pro-inflammatory environment by increasing SPM receptors, an SPM biosynthetic enzyme (15-LOX2) and PPARγ in the AD brain. Also, this study suggests that measuring SPMs in CSF has potential for AD diagnosis and monitoring disease progression. Another study by Emre et al. (2020), reported a comprehensive evaluation of the relationship of AD pathology including early onset AD and MCI, and the distribution of SPM receptors in AD brains. Similar to the findings of Wang et al. (2015b), the BLT1 (LTB4 receptor, and partial agonist for RvE-1) and ChemR23, EPA-derived RvE-1 receptors, were expressed in neurons, astrocytes and microglia of the hippocampal regions, basal forebrain and cingulate gyrus, with higher expression in the AD brains as compared to healthy controls. The receptor expression correlated well with inflammatory markers in microglia and astrocytes, as well as with Braak staging, a measure of disease pathology. Given these findings, and variable expression pattern of these two receptors throughout the brain, the authors hypothesized that these two receptors might modulate neuroinflammation in response to both pro-inflammatory and resolution mediators during both the acute and resolution phases of inflammation.

In a follow-up study by the same group (Zhu et al., 2016), SPMs derived from ARA, EPA, and DHA were detectable in the entorhinal cortex (ENT) brain region which is important for memory and thus, important for AD. While ARA-derived lipoxins did not differ between controls and AD brains, RvD5, MaR1, and protectin D1 (PD1) were lower in AD brains than in controls. Also, the pro-inflammatory PGD2 levels were higher in the AD brains as compared to controls. Direct effects of LXA4, RvD1, and PD1 on cell viability and cell survival were tested on neuroblastoma cells. All tested SPMs were effective in improving cell viability and survival. In Aβ42 stressed/activated microglia, treatment with MaR1 resulted in reduction of Aβ42-induced increase in Cd11b (causes hyper-adhesion) and CD40, respectively. Moreover, MaR1 but not RvD1, PDX (isomer of PD1), or LXA4 increased phagocytosis of Aβ42 in human microglial cells. In addition, SPM receptors GPR32 and ALX/FPR2 were found to be expressed in differentiated neuroblastoma and microglial cells in this study. Another study by Zhao et al. (2012) assessed the role of neuroprotection D1 (NPD1), a lipid mediator derived from DHA in AD pathology. Like LXA4 and LTB4, NPD1 is also reduced in AD brain. Its pro-resolving activity in AD pathology appears to alter Aβ42 release from aging brain cells. To understand if NPD1 is one of the mechanisms through which DHA exhibits its neuroprotective and anti-inflammatory effects in the brain, Zhao et al. (2012) used a transgenic mouse model of AD (PS1, APP, and tau-transgenic) as well as Aβ42 treated aging human neuronal-glial (HNG) co-culture. Age-related decreases were found in hippocampus for both DHA and NPD1 in AD mice as compared to age-matched controls (4 months vs. 12–13-month-old). Similarly, in co-culture experiments with HNG, NPD1 showed neuroprotective effects by reducing cytotoxicity and apoptosis in Aβ42 stressed cells and by down-regulating pro-inflammatory and pro-apoptotic genes [cyclooxygenase-2 (COX-2), tumor necrosis factor-α (TNFα) and B94] while improving cell survival. In addition, NPD1 altered the beta-amyloid precursor (βAPP) protein processing in a dose-dependent manner and stimulated a non-amyloidogenic pathway. The effects of NPD1 on this βAPP processing was in part mediated by modulation of the activity of secretase enzymes (i.e., increase in ADAM10 and decrease in BACE1), which are key enzymes in this process and this effect of NPD1 was dependent on PPARγ.

Yin et al. (2019), investigated the effects of DHA-derived SPM MaR1 in AD. Using a model with Aβ42 infusion into the brain followed by MaR1 injections, they found that treatment with MaR1 reduced the Aβ42-induced increase in pro-inflammatory cytokines and improved AD pathology at the cellular (reduced activation of microglia and astrocyte) and behavioral level (improved learning and memory deficits as measured by escape latency in Morris Water Maze test). Furthermore, MaR1 treatment resulted in upregulation of PI3K/AKT, ERK survival pathway and inhibition of pro-inflammatory proteins, apoptosis, and autophagy, all of which are consistent with effective resolution of inflammation.

Lee et al. (2020), examined the mechanisms of how aspirin-triggered SPM generation occur in a murine model of AD. In their study, they found that a serine residue on COX-2 is acetylated by N-acetyl sphingosine (N-As), and in turn this results in SPM generation. In their *in vivo* model, they found that microglia have reduced levels of N-As and subsequent reduced generation of SPM. These results were further corroborated by a rescue experiment where they treated AD mice with N-As, which increased acetylated COX-2, SPM generation and increased phagocytosis by microglia. Resolution of inflammation in the brain further improved cognitive function in these mice.

RvD1 prevents cognitive decline in animal models (Koivisto et al., 2014), improves Aβ phagocytosis and regulates inflammation in patients with AD (Mizwicki et al., 2013; Fiala et al., 2015; Wang et al., 2015b). Mizwicki et al. (2013), conducted a case control study where they assessed the role of nutraceutical products, DHA-derived RvD1 and vitamin D3 (1, 25-dihydroxyvitamin D3) in peripheral blood mononuclear cells (PBMCs) of healthy subjects (*n* = 3) and AD patients (*n* = 5). Their hypothesis was based on previous evidence showing that microglia and associated phagocytosis of Aβ are dysfunctional in AD patients, and vitamin D3 and RvD1 can genetically modulate macrophage response and improve phagocytosis, respectively (Menegaz et al., 2011; Fiala et al., 2015). Treatment of PBMCs with RvD1 and vitamin D3, resulted in a concentration dependent increase in phagocytosis, and decrease in apoptosis in AD macrophages. These changes occurred via specific receptors, that is GPR32 and protein disulfide isomerase A3 receptors for RvD1 and vitamin D3, respectively. Despite the low number of patients in this study, evaluation of 84 inflammatory genes using a RT2 Profiler (inflammatory/autoimmune gene array) in PBMCs separated the patients into two distinct groups, group 1 with mainly decreased toll-like receptors (TLRs) and Interleukin (IL) Receptor (IL1R1); group 2 with increased transcription of TLRs and cytokines. Regardless of the TLR status, IL1RN, ITGB2 (integrin B2) and nuclear factor kB (NFκB) genes were upregulated, and some of the chemokines and their receptors chemokine ligand (CCL)2, CCL7, CCR1, and IL6R were downregulated as compared to controls, which revealed a unique genetic signature that can serve as a biomarker for isolated AD. Also, when different forms of Aβ proteins were tested on these PBMCs, there was an overall significant upregulation of inflammatory gene transcription in both group 1 and group 2 patients with slight differences in the presence of different Aβ forms as compared to healthy control PBMCs. Both RvD1 and vitamin D3 treatment showed distinct effects on Aβ-induced inflammatory gene expression for group 1 and 2 patients as compared to controls. Both RvD1 and vitamin D3 also decreased sAβ_42_ stimulated increase in cytokines (vitamin D3 was more potent than RvD1) in one of the group 2 patients. Overall, this study showed that RvD1 and vitamin D3 modulates inflammation and macrophage function in AD PBMCs and have potential as treatment improving macrophage phagocytosis of Aβ proteins in AD.

Hopperton et al. (2018), investigated the role of elevated brain levels of n-3 PUFAs on neuroinflammation and AD pathology using *fat-1* transgenic mice that can convert n-6 PUFAs to n-3 PUFAs, and wild-type mice fed either an n-3 (2% fish oil) rich or 10% safflower oil rich diet (low n-3, high n-6 PUFA). In their study, mice were infused with the amyloid β 1–40 peptide to induce neuroinflammation and AD pathology. They found that the amyloid peptide infused mice that were receiving the safflower oil but not the mice receiving the fish oil supplementation had the gene expression signature associated with increased inflammation. In contrast to other studies discussed here, this study was not able to find any differences in eicosanoids in response to different diets or to amyloid β infusion, and could not detect protectin, maresin and D- and E-series SPMs derived from n-3 and n-6 PUFAs. However, some other metabolites that are precursors to SPM were detected. For example, 4-HDHA, an autoxidative product derived from DHA, was found to be higher in the hippocampus of *fat-1* mice as compared to WT mice receiving the safflower oil diet. An EPA-derived 17(18)-EpETE metabolite was found to be elevated in WT mice receiving the fish oil diet, and its less active metabolite 17(18)-DiHETE was found to be elevated in both *fat-1* and WT mice receiving the fish oil diet as compared to WT mice receiving the safflower oil diet. The authors hypothesized that the 17(18)-EpETE metabolite might be protective against AD due to its vasodilatory and PAPRγ mediated actions.

## Specialized Pro-Resolving Lipid Mediators in Parkinson’s Disease

PD is a progressive nervous system disorder characterized by dopaminergic (DA) neuron loss in the substantia nigra (SN) (Hughes et al., 1992). Clinical symptoms include tremor, rigidity and bradykinesia (Levy et al., 2009), while the histopathological hallmark includes the presence of α-synuclein (α-syn)-rich Lewy bodies in neurons of the CNS (Lee and Trojanowski, 2006). Both experimental and clinical studies have reported that neuroinflammation and oxidative stress markedly contribute to the etiology of PD (Wang et al., 2015a; Chen et al., 2016; Rai et al., 2016, 2017a,2017b; Niranjan, 2018). Harmful inflammatory mediators have been found to deteriorate DA neurons (Stoll et al., 2000; Sawada et al., 2006) and aggregated α-syn can induce microglial activation or proliferation and inflammatory cytokine release in PD (Zhang et al., 2005; Su et al., 2008; Beraud et al., 2013) even earlier than the occurrence of DA cell death (Zhang et al., 2005; Beraud et al., 2013). Recently, research has shown that specific, bioactive compounds are able to reduce these deleterious effects in PD models (Singh et al., 2018; Rai et al., 2019; Zahra et al., 2020). SPM may be beneficial in patients with PD by preventing or inhibiting chronic neuroinflammation and preserving DA neurons.

Xu et al. (2013) demonstrated the ability of DHA-derived SPM to ameliorate the pro-inflammatory response of lipopolysaccharide (LPS)-induced murine microglial cells. ELISA and RT-PCR showed pretreatment with RvD1 significantly inhibited NO, TNF-α, and IL-1β and TNF- α, IL-1β, and inducible nitric oxide synthase (iNOS) mRNA expression, respectively. Further, RvD1 inhibited the activity of NFκB, 1 (AP-1) DNA binding and prevented the activation of phosphorylation of mitogen-activated protein kinase (MAPK) signaling pathways extracellular signal-related kinase (ERK)1/2 and p38 MAPK. In another study, the ability of RvD1 to inhibit the progression of an experimental model of PD by suppressing inflammation was investigated by Xu et al. (2017). Here, different RvD1 concentrations (50, 100, and 200 nM) were administered prior to 1-methyl-4-phenylpyridium ion (MPP+) treatment in an *in vitro* PD model (PC12 cells). MPP^+^ affects dopamine neuron loss by entering nigrostriatal neurons by way of dopamine transporters and impacts SN neuronal loss (Birla et al., 2019; Singh et al., 2020). Fluorescein (FITC) and MTT assay revealed RvD1 (100 and 200 nM) dose-dependently inhibited upregulation of PC12 apoptosis and cellular damage. Consistent with previous research in microglial cells, RvD1 inhibited elevation of TNF-α in the PC12 model and inhibited p-P38, p-ERK, and NF-kB p50 signaling pathways (Xu et al., 2013).

Tian et al. (2015) assessed the impact of an n-3-derived lipid mediator on substantia nigra pars compacta (SNpc) *in vivo* on an LPS-induced animal model and on pro-inflammatory cytokine expression *in vitro* in microglia. The group first aimed to evaluate the development of behavioral defects, neuronal damage in the nigrostriatal dopaminergic system. Following LPS injection, Sprague Dawley (SD) rats received different concentrations of intrathecal RvD2 injections (25, 50, or 100 ng/kg) for 27 days. RvD2 treatment significantly decreased apomorphine-elicited rotational cycles, with rotational cycles decreasing with increased SPM dose. RvD2 also inhibited the tyrosine hydroxylase (TH)-positive neurons loss of the SN in LPS-treated rats. In a separate experiment, pretreatment of LPS-induced microglial cells (CD11b +) with RvD2 (2.5, 5, 10, 20 μmol/L) significantly inhibited activation in the SNpc by 19, 25, and 43% with increasing dose. Further, RvD2 was able to inhibit the TLR4/NFκB signaling pathway as well as NFκB p65 mRNA expression by reducing the nuclear translocation of NFκB p65 in microglial cells. Further, RT-PCR revealed RvD2 significantly inhibited pro-inflammatory factors, mRNA expression and reduced level of intracellular ROS as shown by fluorescence microscopy.

Given the relationship between neuroinflammation and α-syn and that the presence of α-syn is pathogenic in PD (Zhang et al., 2005; Beraud et al., 2013). Krashia et al. (2019) investigated the effects of DHA-derived SPM in a bacterial artificial chromosome (BAC) transgenic gene model (Syn rats) that overexpresses human α-syn. Syn rats displayed features common in PD, such as progressive DA cell loss, reduced striatal DA outflow and motor deficits. Neuroinflammation was also present, as evidenced by cytokine production and region-specific microglia activation in the midbrain, striatum and hippocampus. To evaluate if these neuroinflammatory changes were associated with changes in inflammation resolution, levels of RvD1 and RvD2 were assessed in the CSF and plasma of Syn rats. Compared with age-matched controls, CSF levels of RvD1, but not RvD2, were higher in 4-month-old Syn rats. The increase was age dependent compared with 2-month-old Syn rats and was coupled with a corresponding decrease in plasma SPM. When levels were measured in 18-month-old rats, CSF and plasma levels of RvD1 were lower compared with age-matched controls. In an attempt to observe the ability of SPM to prevent neuronal and motor deficits secondary to chronic inflammation, RvD1 (i.p.0.2 μg/kg) was injected into Syn rats twice per week for 8 consecutive weeks starting at 2 months of age. In this experiment, early and chronic SPM treatment prevented changes in plasma IFN-γ levels, microglia activation and peripheral monocyte levels, while also preventing functional deficits and motor impairments (Krashia et al., 2019). Treated animals displayed normal crossing into the center zone in an open field test and improved performance on the accelerating rotarod compared to saline-treated animals, providing evidence that the RvD1 pathway can efficiently prevent neurophysiological and motor defects in Syn rats. In addition, Krashia et al. (2019) also examined levels of SPM (RvD1 and RvD2) and cytokines in the CSF and plasma of early PD patients (*n* = 8) and healthy age-matched controls (*n* = 8). In one of the only studies examining SPM levels in humans, patients diagnosed with PD (symptom duration 13 ± 5 months, untreated and mildly affected) showed significantly higher levels of IL-4 in the CSF and IFN-γ and IL-10 in the plasma compared with controls. Interestingly, levels of RvD1 in both the CSF and plasma were significantly lower (*p* = 0.049 and *p* = 0.007, respectfully). However, no differences in RvD2 were detected between groups.

## Discussion

Both AD and PD preclinical studies discussed here suggest a dysregulation of the SPM levels and their receptors in disease condition as compared to healthy individuals. In both conditions, several studies suggest a benefit from administration of SPMs in various models (Table 1). SPMs hold great promise for neuroprotection in AD by altering gene expression of pro-inflammatory genes, modulating macrophage function, serving as a biomarker for AD status, and promoting resolution of neuroinflammation. In PD, data from *in vitro*, *in vivo*, and observational studies suggest SPM are able to cross the blood-brain barrier, inhibit microglial activation and decrease induced markers of inflammation, possibly as a result of their ability to downregulate NFκB signaling pathways (Xu et al., 2013, 2017; Tian et al., 2015). Further, treatment with these lipid-derived mediators may improve behavioral deficit as a result of the protection of DA neurons and prevents the onset of PD by attenuating neuroinflammation.

**TABLE 1:** Impact of PUFA-derived lipid mediators on neuroinflammation in *in vitro* and *in vivo* neurodegenerative disease models.

Author	Model	PUFA-derived mediators	Effect on neuroinflammation
Wang et al. (2015b)	Post-mortem brain tissue (no neuroinflammation model)	N/A	N/A
	Aβ40-exposed PBMCs	SPM Substrate (1.7 g DHA, 0.6 g EPA)	N/A
Emre et al. (2020)	Post-mortem brain tissue (no neuroinflammation model)	N/A	N/A
Zhu et al. (2016)	STS-induced apoptosis in neuroblastoma cells	LXA4	Improvement
		MaR1	Improvement
		RvD1	Improvement
		PDX	Improvement
	Aβ42 phagocytosis in human microglia	LXA4	No effect
		MaR1	Improvement
		RvD1	No effect
		PDX	No effect
	Aβ42 stressed/activated microglia	MaR1	Improvement
Zhao et al. (2012)	Aβ42-treated HNG	NDP1	Improvement
Yin et al. (2019)	Aβ42-treated C57BL/6 mouse hippocampus	MAR1	Improvement
Lee et al. (2020)	Murine AD	Aspirin-triggered SPM	Improvement
Koivisto et al. (2014)	Microglia activation of APP/PS1 Mice	SPM Substrate (fish-oil based diet)	Improvement
Fiala et al. (2015)	Aβ42-exposed PBMCs	SPM Substrate, RvD1 (1 g DHA, 1 g EPA)	Improvement
Mizwicki et al. (2013)	AD patient PBMCs	RvD1 + Vitamin D3	Improvement
Hopperton et al. (2018)	Aβ40-exposed mice	SPM Substrate (fish-oil based diet)	Improvement
Xu et al. (2013)	LPS-induced murine microglial cells	RvD1	Improvement
Xu et al. (2017)	MPP + treated PC12	RvD1	Improvement
Tian et al. (2015)	LPS-induced SD rats	RvD2	Improvement
	LPS-induced CD11b +	RvD2	Improvement
Krashia et al. (2019)	Syn Rats	RvD1	Improvement

*PUFA, Polyunsaturated Fatty Acid; 3xTg-AD mice, harboring PS1 (M146V), APP (Swe) and tau (P301L) human transgenes; Aβ40, amyloid beta 1-40; PBMC, Peripheral blood mononuclear cells; SPM, Specialized pro-resolving mediator; DHA, Docosahexaenoic acid; EPA, Eicosapentaenoic acid; STS, Staurosporine; LXA4, Lipoxin A4; MaR1, Maresin-1; RvD1, Resolvin D1; PDX, Protectin D Isomer; Aβ42, Amyloid Beta 1–42; HNG, human neuronal-glial co-culture; NDP1, Neuroprotectin D1; AD, Alzheimer’s disease; LPS, Lipopolysaccharide; MPP+, 1-methyl-4-phenylpyridium; SD, Sprague Dawley; RvD2, Resolvin D2.*

Many *in vitro* systems, including immortalized cell lines, patient derived iPSCs or iPSC-derived cell lines, and organoids have been used in studying neurodegenerative disorders. Some of the main problems of using *in vitro* cultures for the study of neurodegenerative disorders is the lack of blood brain barrier, interactions between brain cells resulting from the growth of cells as 2D monolayers (Konnova and Swanberg, 2018). These problems were overcome by using 3D cultures, organoids, scaffolds, microfluidics platform to mimic blood flow etc. Cell systems mimicking the blood-brain barrier has been recently reviewed by Peng et al. (2021). Neuroinflammation associated with neurodegeneration can be mimicked by more advanced 3D glial cell culture systems as well as co-culture of microglia, neuroblastoma and endothelial cells (Tu et al., 2019; Yin et al., 2020). These 3D systems can recapitulate the extracellular aggregation of amyloid beta as in AD (Choi et al., 2016) and aggregation of alpha-synuclein in neurons differentiated from iPSCs derived from PD patients (Oh, 2019).

To study the pathology and progression of neurodegenerative disorders, mouse models have been established using neurotoxin (environmental toxins, rotenone and paraquat, MPTP, 6-OHDA) and genetic approaches (knockout mouse models and conditional knockout mouse models) (Konnova and Swanberg, 2018); however, none of these murine models seem to capture all features of disease. Considering the mouse models also vary, it is not always possible to extrapolate results from one model to another. One of the important differences where the effects of treatments as well as novel treatments like SPM might differ is the varying degree of neuroinflammation in these models. Studies have shown that neuroinflammation has an important role in the pathogenesis of neurodegenerative diseases regardless of genetic or toxin-induced models, as evidenced by the presence of activated microglia in postmortem SN, as well as increased levels of pro-inflammatory cytokines in the CSF and serum of patients (Wang et al., 2015b; Zhu et al., 2016; Krashia et al., 2019). Supporting this evidence, injection of LPS into SN has been shown to induce neurotoxicity, activate microglia and cause neurodegeneration in DA and non-dopaminergic neurons (Tufekci et al., 2011). Litteljohn et al. (2010) compared different toxin-induced PD models. These toxins have differences in their mechanisms of action, yet all lead to the loss of DA neurons. The timing of SPM treatment is another issue that needs to be addressed in preclinical studies. For example, their prophylactic effects as well as therapeutic effects need to be addressed in mouse models of disease. One of the challenges of AD and PD is that patients show variability in their symptoms, age of diagnosis, as well as disease progression.

Minimal data evaluating these PUFA-derived metabolites in humans with neurodegenerative disorders are available and a gap in knowledge exists regarding behavior of SPM and their receptors in patients with these conditions. There is also large gap in our knowledge regarding which SPMs would be most effective in which model of AD or PD and how dietary intake or supplementation can impact SPM levels. Previous work suggests PUFA supplementation may increase plasma levels of lipid-derived mediators in humans under a stressed state (Nordgren et al., 2019). However, there is a current paucity of data regarding baseline SPM levels in patients with neurodegenerative disorders as well as amount of substrate needed to result in a potential clinical improvement. One way to approach these issues is to determine SPM levels in different stages of the disease in both AD and PD, which might be addressed in preclinical or clinical studies. Further, as growing evidence demonstrates overlapping clinical and neuropathologic features of the two conditions, the role of SPM in resolving inflammation in Parkinson’s dementia should also be investigated. As of now, there are no human studies looking into differences in individuals regarding SPM receptor expression or which SPMs would be most helpful to reduce neuroinflammation in AD patients. Furthermore, SPMs affect microglia activation, which can change the course of events from pro-inflammatory to anti-inflammatory depending on the M1/M2 polarization status, and this can lead to neurotoxic or neuroprotective effects. Despite the differences in major symptoms (memory vs. physical), similar issues related to SPMs are also relevant in PD due to the common neuroinflammatory mechanisms. In both diseases, the long-term effects of SPM supplements should be tested to uncover any unfavorable effects or any repair pathways that might be affected by SPM treatment. To the best of our knowledge, there are limited studies investigating the roles of SPMs in AD or PD, and no studies report any adverse effects of SPMs in these disease states. Nevertheless, some studies report that some survival pathways are activated, for example PI3K/Akt survival pathway, which is a pathway that is also implicated in cancers and resistance in cancer therapy. Therefore, the nuances of SPM treatment would need to be defined in AD and PD patients once it acquires clinical use. Large epidemiological studies seeking to understand the effect of diet on SPM levels would be most useful. The ability of SPM to prevent or resolve chronic neuroinflammation may represent a novel approach to treatment. Future direction should include focused, translational efforts to investigate SPM as an add-on (in addition to standard treatment) or as standalone agents in patients with neurodegenerative disorders.

## Author Contributions

JP, AU, CH, and DB: conceptualization. JW, AU, and CH: methodology. DB, JB, EC-S, AF, K-CS, VM, and JA: validation. JP and AU: investigation, data curation, and writing—original draft preparation. DB, JB, CH, JW, EC-S, AF, K-CS, VM, and JA: writing—review and editing. JP, AU, and CH: visualization. DB: supervision and project administration. All authors contributed to the article and approved the submitted version.

## Conflict of Interest

The authors declare that the research was conducted in the absence of any commercial or financial relationships that could be construed as a potential conflict of interest.

## Publisher’s Note

All claims expressed in this article are solely those of the authors and do not necessarily represent those of their affiliated organizations, or those of the publisher, the editors and the reviewers. Any product that may be evaluated in this article, or claim that may be made by its manufacturer, is not guaranteed or endorsed by the publisher.
